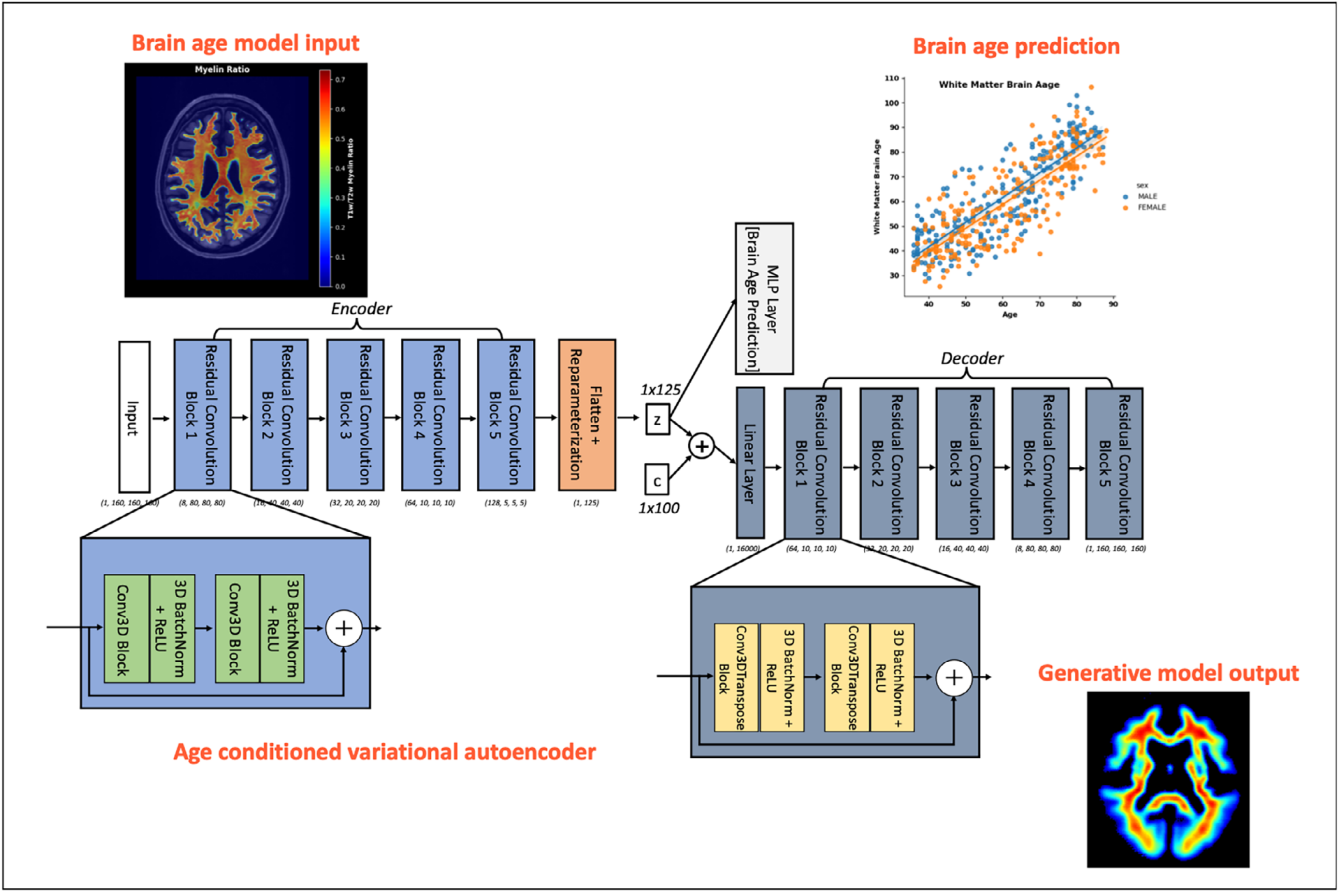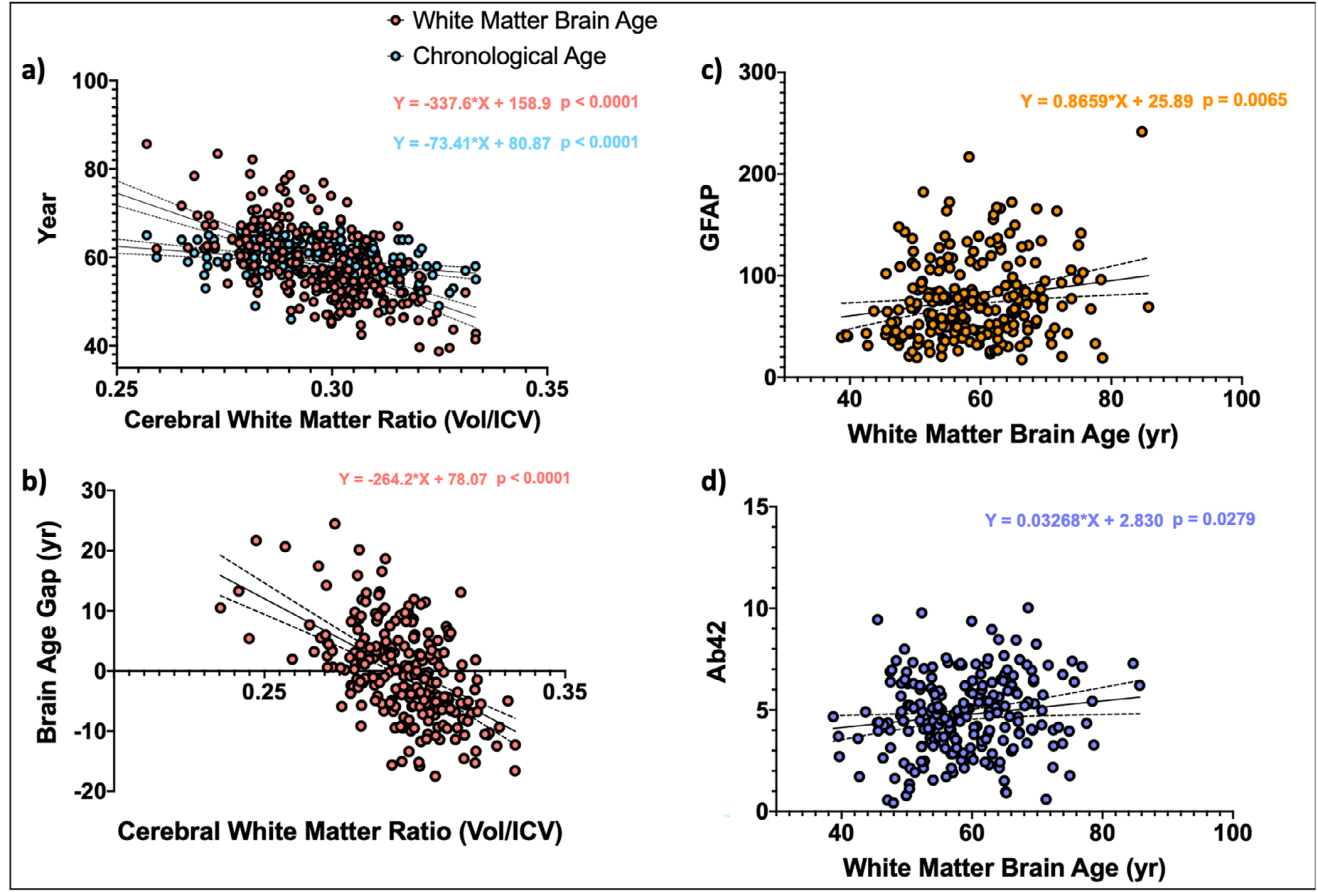# White matter aging in late midlife women: Validation and association with neurodegenerative biomarkers

**DOI:** 10.1002/alz.092818

**Published:** 2025-01-09

**Authors:** Jinghang Li, Minjie Wu, Chang‐Le Chen, Linghai Wang, Shaolin Yang, Howard J Aizenstein, Yuefang Chang, Thomas K Karikari, Carol A. Derby, Pauline M Maki, Rebecca C Thurston

**Affiliations:** ^1^ University of Pittsburgh, Pittsburgh, PA USA; ^2^ Department of Bioengineering, University of Pittsburgh, Pittsburgh, PA USA; ^3^ Department of Psychiatry, School of Medicine, University of Pittsburgh, Pittsburgh, PA USA; ^4^ Department of Neurosurgery, University of Pittsburgh, Pittsburgh, PA USA; ^5^ Department of Neurology, and Department of Epidemiology and Population Health, Albert Einstein College of Medicine, Bronx, NY USA; ^6^ Department of Psychiatry, University of Illinois at Chicago, Chicago, IL USA

## Abstract

**Background:**

Brain age (BA) prediction models have emerged as valuable tools for understanding individual differences in trajectories of brain aging. These models aim to estimate overall brain health by predicting BA based on structural MRI data. To enhance the specificity of existing BA models, we introduce a deep learning‐based BA prediction model. The model focuses on the cerebral white matter to assess the prominent cerebrovascular AD risk pathway for women. Here we validate our deep learning‐derived white matter brain age (WMBA) in a sample of late midlife women enrolled in MsBrain. We also examine WMBA in relation to plasma neurodegenerative biomarkers in this sample.

**Method:**

We developed an age‐conditioned variational autoencoder along with a multilayer perceptron (MLP) for brain age prediction (Figure 1). The model was trained on the Human Connectome Project healthy aging (HCP‐A) dataset (female, n=396; male, n=316, 36‐90 yr, mean=60 yr). To achieve white matter specificity, the model was trained only on the myelin ratio map. Adaptability and credibility were assessed by testing the model with MsBrain data, a study for which midlife women with no cardiovascular disease, stroke, or dementia were recruited (n=239, 45‐67 yr, mean=59 yr). Plasma biomarkers included amyloid β (aβ) 42, phosphorylated tau (181, 231), glial fibrillary acidic protein (GFAP), and neurofilament light (NFL) via Simoa. All brain age predictions underwent bias correction through fitted linear regression models.

**Result:**

Figure 1 shows the overall WMBA model schematics, including myelin ratio map training inputs, brain age predictions, and generated WMBA outputs. Compared with chronological age, the WMBA model was more strongly associated with cerebral white matter volume (Figure 2a). When considering brain age gap (i.e., difference between predicted brain age and chronological age), a greater brain age gap (indicating older brain age) was found with lower cerebral white matter ratio (Figure 2b). Lastly, greater WMBA was associated with higher GFAP and aβ42.

**Conclusion:**

This work validates a new approach for measuring WMBA and demonstrates its utility in studies of midlife women. Findings indicate that WMBA varies with plasma markers of neurodegeneration in late midlife women, suggesting a novel neuroimaging marker to detect early neurodegenerative changes.